# A recessive allele for delayed flowering at the soybean maturity locus *E9* is a leaky allele of *FT2a*, a *FLOWERING LOCUS T* ortholog

**DOI:** 10.1186/s12870-016-0704-9

**Published:** 2016-01-19

**Authors:** Chen Zhao, Ryoma Takeshima, Jianghui Zhu, Meilan Xu, Masako Sato, Satoshi Watanabe, Akira Kanazawa, Baohui Liu, Fanjiang Kong, Tetsuya Yamada, Jun Abe

**Affiliations:** Research Faculty of Agriculture, Hokkaido University, Sapporo, Hokkaido 060-8589 Japan; Faculty of Agriculture, Saga University, Saga, 840-0027 Japan; The Key Laboratory of Soybean Molecular Design Breeding, Northeast Institute of Geography and Agroecology, Chinese Academy of Sciences, Harbin, 150081 China

**Keywords:** Maturity gene *E9*, *FLOWERING LOCUS T*, *FT2a*, Soybean (*Glycine max*), Flowering, *Ty1/copia*-like retrotransposon, *SORE-1*, Methylation

## Abstract

**Background:**

Understanding the molecular mechanisms of flowering and maturity is important for improving the adaptability and yield of seed crops in different environments. In soybean, a facultative short-day plant, genetic variation at four maturity genes, *E1* to *E4*, plays an important role in adaptation to environments with different photoperiods. However, the molecular basis of natural variation in time to flowering and maturity is poorly understood. Using a cross between early-maturing soybean cultivars, we performed a genetic and molecular study of flowering genes. The progeny of this cross segregated for two maturity loci, *E1* and *E9*. The latter locus was subjected to detailed molecular analysis to identify the responsible gene.

**Results:**

Fine mapping, sequencing, and expression analysis revealed that *E9* is *FT2a*, an ortholog of *Arabidopsis FLOWERING LOCUS T*. Regardless of daylength conditions, the *e9* allele was transcribed at a very low level in comparison with the *E9* allele and delayed flowering. Despite identical coding sequences, a number of single nucleotide polymorphisms and insertions/deletions were detected in the promoter, untranslated regions, and introns between the two cultivars. Furthermore, the *e9* allele had a *Ty1/copia*–like retrotransposon, *SORE-1*, inserted in the first intron. Comparison of the expression levels of different alleles among near-isogenic lines and photoperiod-insensitive cultivars indicated that the *SORE-1* insertion attenuated *FT2a* expression by its allele-specific transcriptional repression. *SORE-1* was highly methylated, and did not appear to disrupt *FT2a* RNA processing.

**Conclusions:**

The soybean maturity gene *E9* is *FT2a*, and its recessive allele delays flowering because of lower transcript abundance that is caused by allele-specific transcriptional repression due to the insertion of *SORE-1*. The *FT2a* transcript abundance is thus directly associated with the variation in flowering time in soybean. The *e9* allele may maintain vegetative growth in early-flowering genetic backgrounds, and also be useful as a long-juvenile allele, which causes late flowering under short-daylength conditions, in low-latitude regions.

**Electronic supplementary material:**

The online version of this article (doi:10.1186/s12870-016-0704-9) contains supplementary material, which is available to authorized users.

## Background

Knowledge of molecular mechanisms of flowering and maturity is important for understanding the phenology of seed crops and for maximizing yield in a given environment. On the basis of knowledge accumulated for *Arabidopsis thaliana*, the molecular mechanisms of flowering have been studied in many crops. These studies have revealed common important genes, such as *FLOWERING LOCUS T* (*FT*) and *CONSTANS* (*CO*), but also their functional divergence and diversity of genetic mechanisms underlying the natural variation of flowering time within species [1–3].

Soybean (*Glycine max* (L.) Merrill) is a facultative short-day plant. Rich genetic variability in photoperiod responses enables the crop to adapt to a wide range of latitudes. This wide adaptability has been created by natural variations in a number of major genes and quantitative trait loci (QTLs) that control flowering [4]. Ten major genes have been identified so far to control time to flowering and maturity in soybean: *E1* and *E2* [5], *E3* [6], *E4* [7], *E5* [8], *E6* [9], *E7* [10], *E8* [11], *E9* [12], and *J* [13]. Dominant alleles at *E6*, *E9*, and *J* promote early flowering, whereas dominant alleles at other loci delay flowering and maturity. *E6* and *J* have been identified in the progeny of crosses between standard and late-flowering cultivars with a long-juvenile habit, which causes late flowering under short days [9, 13]. *E9* has been identified through the molecular dissection of a QTL for early flowering introduced from a wild soybean accession [12, 14]. Molecular mechanisms that involve four of the ten genes (*E1* to *E4*) have been identified. *E1* encodes a possible transcription factor down-regulating *FT2a* and *FT5a* (soybean *FT* orthologs) [15] and has the most marked effect on flowering time [16–18]. *E2* is an ortholog of *Arabidopsis GIGANTEA* (*GI*) [19]. *E3* and *E4* encode the phytochrome A isoforms, GmPHYA3 and GmPHYA2, respectively [20, 21].

The soybean genome has at least ten *FT* homologs, among which six promote flowering of the *Arabidopsis ft* mutant or ecotype Columbia (Col-0) when ectopically expressed [22–25]. Their expression profiles differ depending on tissues and growth stages, suggesting their subfunctionalization in soybean flowering [23–25]. Among the six homologs, *FT2a* and *FT5a* have been extensively studied [15, 19, 22–28], because their expression patterns closely follow photoperiodic changes [24] and their overexpression promotes flowering even under non-inductive conditions [26, 27]. The photoperiodic expression patterns of *FT2a* and *FT5a* are most likely controlled by *E1* and its homologs, *E1La* and *E1Lb*, which in turn are under the control of *E3* and *E4* [15, 28]. *E2* inhibits *FT2a* expression possibly through a pathway different from the E1–PHYA pathway [19, 28].

Allelic variations at *E1*–*E4* generate some but not all of the variation in flowering time among soybean cultivars [18, 29]. Various combinations of mutations that occur independently at *E1*, *E3*, and *E4* lead to insensitivity or low sensitivity of flowering to photoperiod [29, 30]. Besides the above four genes, a number of soybean orthologs of *Arabidopsis* flowering genes have been characterized: *COL* (*CO-like*) [25, 31], *CRY* (*CRYPTOCHROME*) [32, 33], *FKF1* [34], *FLD* (*FLOWERING LOCUS D*) [35], *FUL* (*FRUITFULL*) [36], *RAV*-*like* (*RELATED TO ABI3/VP1*-*like*) [37], *SOC1*/*AGL20* (*SUPPRESSOR OF OVEREXPRESSION OF COL1/AGAMOUS-LIKE 20*) [38, 39], *TARGET OF EAT1* (*TOE*) [40], and *ZTL* (*ZEITLUPE*) [41]. A genome-wide association study also revealed a number of SNPs that were significantly associated with flowering time; some of these SNPs implied an involvement of orthologs to *Arabidopsis* flowering genes, such as *EARLY FLOWERING 8* and *SOC1* or *AGAMOUS-LIKE 6*, in the control of flowering time in soybean [42]. However, our understanding of the roles of these orthologs in the natural variation of flowering in soybean is still limited. Jiang et al. [43] found diverse sequence variations in the *FT2a* promoter region among soybean cultivars, despite the coding region being highly conserved. Although some of these polymorphisms are significantly associated with variation in flowering time among the cultivars tested, their roles in *FT2a* expression is not fully understood [43].

In this study, using a cross between early-maturing cultivars of different origins, we found that segregation of flowering time was partly associated with a tagging marker of the maturity gene *E9*. We demonstrate that *E9* is identical to *FT2a*, and its recessive allele has an insertion of the *Ty1/copia*-like retrotransposon in the first intron, which reduces the *FT2a* transcript level and delays flowering.

## Results

### Segregation of flowering time in the progeny of a cross between Harosoy and Toyomusume

**Fig. 1:**
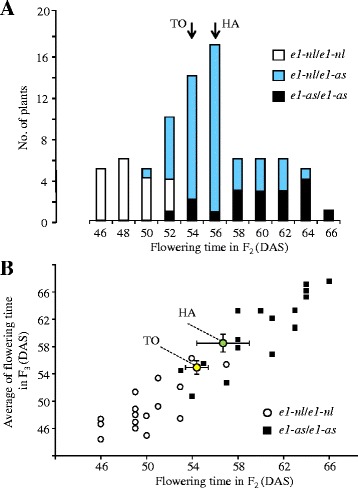
Flowering time in the progeny of the cross between Toyomusume and Harosoy. **a** Frequency distribution of flowering time in F_2_. **b** Scatter diagram of flowering time in F_2_ and F_3_ progeny. Averages and standard deviations of flowering time for Toyomusume (TO) and Harosoy (HA) are shown

Two early-maturing cultivars, a Canadian cultivar, Harosoy (HA), and a Japanese cultivar, Toyomusume (TO), were used in the crossing. They have the same maturity genotypes at *E2*, *E3*, and *E4* (*e2*/*e2 E3*/*E3 E4*/*E4*), but differ in the *E1* genotype: HA has a hypomorphic *e1-as* allele, whereas TO has an *e1-nl* allele, which lacks the genomic region (~130 kb) containing the entire *E1* gene [15, 18]. TO and HA flowered almost at the same time under natural daylength conditions in Sapporo, Japan (43°07′N, 141°35′E), although the former flowered 3 to 5 days earlier than the latter. However, flowering times in the F_2_ population varied widely (46–67 days after sowing; Fig. 1a). Since the allelic variation at *E1* has a large effect on flowering time, we first evaluated the effects of *E1* alleles on flowering time in the population. We determined the *E1* genotypes of F_2_ plants with an allele-specific DNA marker [29] and flanking simple sequence repeat (SSR) markers [15]. As expected, plants homozygous for *e1-nl* (from TO) flowered, on average, 11 days earlier than those homozygous for *e1-as* (from HA) (Fig. 1a). Since plants homozygous for each allele still varied considerably in flowering time, we carried out the progeny test for 16 plants homozygous for each allele. Flowering times of F_2_ individuals were closely correlated with the average flowering times of their progeny (Fig. 1b). Parent–offspring correlation coefficients were 0.676 for the *e1-nl* homozygote and 0.823 for the *e1-as* homozygote, suggesting that a genetic factor(s) other than *E1* segregated in each of the two genotypic classes.

### Test for association between flowering time and SSR markers

To detect flowering genes that segregated independently of *E1*, we tested flowering time–SSR marker association in each of the *e1-nl* and *e1-as* genotypic classes; we used 61 SSR markers located in the genomic regions where orthologs to *Arabidopsis* flowering genes are clustered [4]. Two markers were significantly associated with flowering time in *e1-nl* homozygotes and five in *e1-as* homozygotes (Table 1). Plants homozygous for the TO alleles (A) at all loci except Sat235 flowered later than those homozygous for the HA alleles (B). Only Sat_350 showed significant associations in both *e1-nl* and *e1-as* genotypic classes. Sat_350 was located near the SSR marker Satt686 on LG J, which is a tagging marker for the *E9* gene identified in a cross between cultivated (TK780) and wild (Hidaka 4) soybeans [12]. Because TO is a parent of TK780 [44], which carries the recessive *e9* allele [12], it is plausible that the gene tagged by Sat_350 is identical to *E9* and that TO has the same recessive allele for late flowering as TK780.

**Table 1:** Association tests of SSR marker genotypes with flowering time

Marker	LG	Average of flowering time (DAS) in			One-way ANOVA	
		AA	AB	BB	F value	Probability
*Plants homozygous for e1-nl*						
Satt681	C2	52.6	48.7	48.5	4.6	0.030
Sat_350	J	55.5	49.3	48.0	8.9	0.004
*Plants homozygous for e1-as*						
Satt519	B1	63.7	60.6	56.4	5.9	0.015
Sat_235	C1	54.0	59.2	63.2	3.9	0.047
Satt031	D2	63.3	60.4	56.0	5.0	0.025
Satt146	F	63.5	62.1	56.6	10.1	0.002
Sat_350	J	62.7	60.0	56.4	5.8	0.016

16 plants homozygous for *e1-nl *and 16 plants homozygous for *e1-as*were used in the association tests. A and B indicate the alleles from Toyomusume and Harosoy, respectively

LG, linkage group

### Fine-mapping and association analysis

**Fig. 2:**
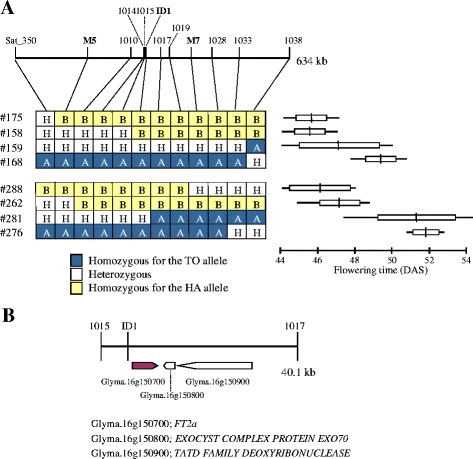
Fine mapping of the *E9* locus and annotated genes in the delimited genomic region. **a** Eight recombinants (four from each of two F_3_ heterozygous plants) in the region between Sat_350 and BARCSOYSSR_16_1038 were genotyped at 7 BARCSOYSSR (1010 to 1033) and 3 indel markers (bold). The genotype at *E9* was estimated by progeny testing. The ranges (horizontal lines), averages (vertical lines), and standard deviations (open boxes) of flowering time (DAS: days after sowing) are indicated. **b** Three annotated genes in a delimited genomic region

For fine-mapping of the *E9* gene, a total of 300 seeds from two heterozygous F_3_ plants derived from the same F_2_ family (#41) were genotyped for the SSR markers Sat_350 and BARCSOYSSR_16_1038. We detected eight recombinants (four progenies from each of two heterozygous F_3_ plants) in the flanking region, which were genotyped for seven additional SSR markers and three insertion/deletion (indel) markers (ID1, M5, and M7) used in the identification of *E9* [12]. The genotype at *E9* was estimated from the segregation pattern in the progeny test (Fig. 2a). Among the four plants derived from one F_3_ parent, two plants (#158 and #175) flowered early and one (#168) flowered late, whereas plant #159 segregated for flowering time. Among the four plants derived from the other F_3_ parent, two plants (#262 and #288) flowered early and one (#276) flowered late, whereas one plant (#281) segregated. By comparing the graphical genotypes and estimated *E9* genotypes, we delimited the QTL to a 40.1-kb region between markers BARCSOYSSR_16_1015 and BARCSOYSSR_16_1017, in which only the ID1 marker completely co-segregated with the genotype at *E9*.

To confirm co-segregation between flowering time and ID1 genotype, we examined 14 F_2_ families homozygous for *e1-nl* and 14 homozygous for *e1-as* (Table 2). Among the *e1-nl* families, plants of two families homozygous for the TO allele flowered late, whereas plants of two families homozygous for the HA allele flowered early. A highly significant association between flowering time and marker genotypes was observed in the 10 heterozygous families. Similarly, a highly significant association was detected between flowering time and marker genotypes in the 5 heterozygous families with the *e1-as* genotype. Therefore, the variation in flowering time in each F_2_ family could be mostly accounted for by the genotypes at the ID1 marker.

**Table 2:** Association tests of ID1, a tagging marker of *E9*, with flowering time

F_2_	Average (SD) of flowering time (DAS) in F_3_			One-way ANOVA	
Plant number	AA	AB	BB	F value	Probability (10^−3^)
F_2_ families with *e1-nl*/*e1-nl*					
#34			44.5 (1.1)		
#66			44.9 (0.8)		
#02	51.8 (2.2)	44.6 (1.4)	44.3 (0.5)	27.2	0.005
#05	53.0 (1.7)	46.3 (1.4)	43.0 (0.8)	45.6	0.000
#25	52.8 (2.8)	47.7 (1.9)	44.8 (1.2)	22.1	0.018
#27	52.6 (2.1)	45.5 (0.8)	44.0 (0.0)	73.2	0.000
#28	56.0 (1.7)	52.1 (1.2)	49.5 (2.4)	14.4	0.223
#41	54.0 (2.0)	46.2 (1.7)	44.2 (1.6)	40.7	0.000
#50	56.0 (1.0)	53.6 (2.1)	50.4 (2.9)	8.8	2.324
#79	56.0 (1.4)	47.8 (1.8)	45.4 (1.3)	61.3	0.000
#81	55.3 (1.6)	46.3 (1.0)	44.7 (0.5)	156.8	0.000
#82	56.4 (0.5)	52.1 (1.0)	46.9 (3.7)	26.2	0.006
#18	55.4 (1.5)				
#46	56.4 (1.5)				
F_2_ families with *e1-as*/*e1-as*					
#12			60.7 (1.9)		
#29			50.7 (1.5)		
#30			52.7 (2.9)		
#43			54.5 (2.4)		
#22	64.8 (1.3)	57.5 (1.6)	52.0 (2.8)	92.0	0.000
#36	65.7 (0.6)	57.3 (3.2)	50.4 (3.8)	57.9	0.020
#48	65.7 (1.6)	59.5 (2.7)	55.0 (1.7)	63.7	0.005
#69	66.7 (1.4)	62.5 (3.8)	59.3 (2.1)	42.6	7.615
#73	65.4 (1.5)	55.6 (2.7)	55.0 (4.0)	58.9	0.015
#13	66.2 (0.8)				
#16	67.4 (0.6)				
#33	63.2 (3.2)				
#76	63.3 (1.9)				
#78	65.9 (0.8)				

The progeny of 14 plants homozygous for *e1-nl* and 14 plants homozygous for *e1-as* were used in the association tests. A and B indicate the alleles from Toyomusume and Harosoy, respectively

### cDNA sequencing and expression analysis

**Fig. 3:**
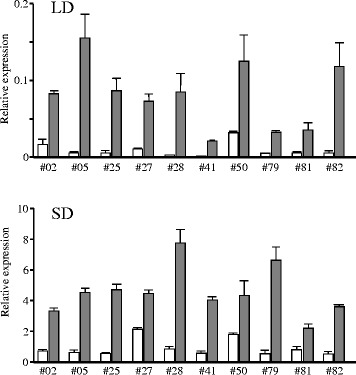
*FT2a* expression in the progeny of F_2_ plants from a cross between Toyomusume and Harosoy. Four plants from the progeny of each F_2_ plant, which were homozygous for the Toyomusume allele (white bars) or the Harosoy allele (gray bars) at the ID1 tagging marker for *FT2a*, were used. Relative mRNA levels are expressed as the ratios to β-tubulin transcript levels

According to the Williams 82 reference genome sequence [45], the region delimited by fine mapping contained three genes: Glyma.16 g150700 (*FT2a*), Glyma.16 g150800 (*EXOCYST COMPLEX PROTEIN EXO70*), and Glyma.16 g150900 *(TATD FAMILY DEOXYRIBONUCLEASE*) (Fig. 2b). We focused on *FT2a* as a candidate for *E9* because of its importance in floral induction in soybean [22–28, 43]. cDNA sequence analysis was carried out for HA and TO, the Japanese cultivar Hayahikari (HY), and the parents (TK780 and Hidaka 4) of the recombinant inbred line (RIL) population used for the identification of *E9* [12]. There were no nucleotide substitutions in their coding regions, which were identical to that of Williams 82; a SNP (#28; Additional file 1) after the stop codon was identified between HA and TO or HY. We then compared the expression profiles of *FT2a* under short day (SD) and long day (LD) conditions in plants homozygous for the TO allele and those homozygous for the HA allele at ID1 in the progeny of 10 F_2_ families with the *e1-nl*/*e1-nl* genotype that segregated for *E9*. The *FT2a* transcript abundance was analyzed at Zeitgeber time 3. In all tested families, plants with the HA allele had higher *FT2a* expression than plants with the TO allele, regardless of daylength, although the expression was much higher in SD than LD in both homozygotes (Fig. 3). The lower expression of *FT2a* in plants with the TO allele was further confirmed in the diurnal expression patterns in TO and HA: the expression levels of TO were very low across any sampling times compared with that of HA (Additional file 2). Thus, late flowering in plants homozygous for the TO allele at ID1 was tightly associated with reduced *FT2a* expression.

### Sequence analysis of the *FT2a* genomic region

In *Arabidopsis*, *FT* is regulated by various transcription factors, which bind to the promoter or to the first intron and 3′ downstream region [1, 3]. To detect the cause of the reduced *FT2a* expression, we first sequenced the 5′-upstream region of *FT2a* in the three cultivars and in TK780 and Hidaka 4. We detected 8 SNPs and 6 indels. The sequences of TO and TK780 were identical to each other, but differed from those of HA and Hidaka 4 in a 43-bp indel in the promoter and a 10-bp indel in the 5′ UTR, which were located 731 and 47 bp upstream of the start codon, respectively, and in two SNPs (#2 and #4) (Additional file 1). The sequence of HY was similar to those of TO and TK780 (including the 43-bp segment), but differed from them in one SNP (#1), a 4-bp indel 274 bp upstream of the start codon, and the 10-bp indel in the 5′ UTR.

We also sequenced the introns and the 3′-downstream region in TO, HA, and HY to test whether the polymorphism(s) observed in the promoter and 5′ UTR could be responsible for late flowering in TO. The primers based on the gene model Glyma.16 g150700 worked well for PCR amplification of these regions except for the first intron of TO. To sequence the first intron in TO, we used genome walking. Nested PCR analysis of genomic libraries produced an amplicon of 370 bp from the library constructed by using *Eco*RV. Sequencing revealed that it consisted of an unknown sequence of 137-bp fused with a 233-bp segment of the first intron of *FT2a* proximal to the second exon. A BLAST search of the NCBI genome database showed that the former sequence was identical to a part of an LTR of *SORE-1* (AB370254), which has been previously detected in a recessive allele at the *E4* locus [21, 46]. The inserted retrotransposon and its flanking regions were then amplified by nested PCR and sequenced. The retrotransposon was 6,224 bp long; its sequence was 100 % identical to the LTRs of *SORE-1* and 99.7 % identical to its coding region. Using a DNA marker for the *SORE-1* detection, we confirmed that TK780 also had *SORE-1* in the first intron, but Hidaka 4, HA, and HY did not. We detected a total of 17 polymorphisms (10 SNPs, 2 indels, and 5 SSRs) from the first intron to 3′ downstream regions among the three cultivars (Additional file 1).

**Fig. 4:**
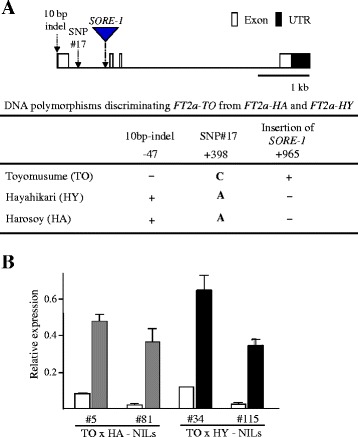
DNA polymorphisms that discriminate between the *FT2a* alleles and *FT2a* transcript abundance in their NILs. **a** Genomic positions and types of three DNA polymorphisms between Toyomusume (TO) and both Harosoy (HA) and Hayahikari (HY) **b** *FT2a* expression in 20-DAS-old plants of NILs for *FT2a-TO *(white) and *FT2a-HA *(gray) or *FT2a-HY *(black) under SD conditions. Relative mRNA levels are expressed as the ratios to β-tubulin transcript levels

Thus, three early-maturing cultivars—TO, HA, and HY—had different *FT2a* sequences, which were designated as the *FT2a*-*TO*, *FT2a*-*HA*, and *FT2a*-*HY* alleles. *FT2a*-*TO* differed from both *FT2a-HA* and *FT2a-HY* in the 10-bp deletion in the 5′ UTR, and in SNP #17 and the *SORE-1* insertion in intron 1 (Fig. 4a, Additional file 1). By using the database of plant *cis*-acting regulatory DNA elements (PLACE) [47], we detected a W-box element (AGTCAAA) that was created by SNP #17 in TO, and two *cis*-elements, RBCSCONSENSUS (AATCCAA) and ARR1AT (NGATT), in the genomic region flanking the *SORE-1* integration site.

### Expression of different *FT2*a alleles in near-isogenic lines and photoperiod-insensitive accessions

We developed four sets of NILs for the above three *FT2a* alleles from the progeny of F_5_ heterozygous plants: two from the cross between TO and HA (#5 and #81) and two from the cross between TO and HY (#34 and #115). We found that, under SD conditions, *FT2a*-*TO* expression was much lower than that of *FT2a*-*HA* and *FT2a*-*HY* (Fig. 4b).

**Fig. 5:**
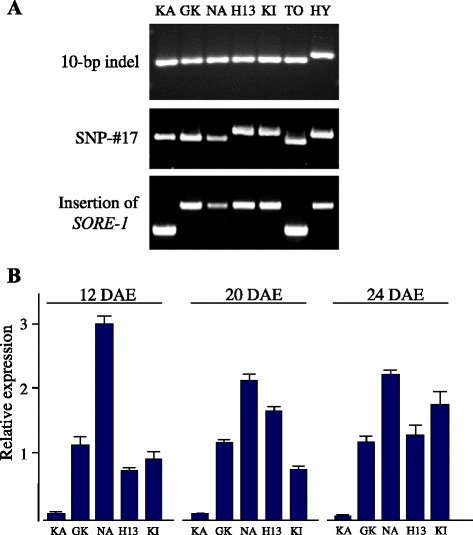
*FT2a* transcript abundance in photoperiod-insensitive *e3 e4* cultivars under SD conditions. **a** DNA polymorphisms in the 10-bp indel, SNP #17, and *SORE-1* insertion. **b** *FT2a* expression at the first (12 days after emergence: DAE), second (20 DAE), and third (24 DAE) leaf stages. Relative mRNA levels are expressed as the ratios to β-tubulin transcript levels. KA, Karafuto 1; GK, Gokuwase-Kamishunbetsu; NA, Napoli; H13, Heihe 13; KI, Kitamusume; TO, Toyomusume; HY, Hayahikari

Using 3 markers, we selected five photoperiod-insensitive *e3 e4* cultivars, all of which had the 10-bp deletion in 5′ UTR, but differed in SNP #17 and in the presence or absence of *SORE-1* (Fig. 5a). We analyzed *FT2a* expression in fully-expanded trifoliate leaves at different leaf stages (first, second, and third true leaves) (Fig. 5b). *FT2a* expression was markedly low in all stages in Karafuto 1, but was relatively high in the other four. Because Karafuto 1 differed from the other cultivars only in the presence of *SORE-1*, low expression of *FT2a*-*TO* was caused by the insertion of *SORE-1*, not by the 10-bp deletion or by SNP #17.

### RNA processing and DNA methylation at the *FT2a* locus

**Fig. 6:**
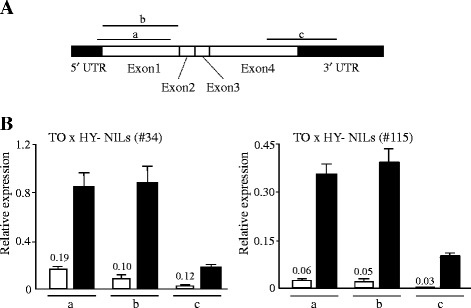
*FT2a* transcript abundance in two sets of NILs for *FT2a-TO* and *FT2a-HY* alleles. **a** Three regions (a-c) in the *FT2a* coding region used to assess transcript abundance. The 5' UTR and 3' UTR are a part of exon 1 and exon 4, respectively. **b** *FT2a* expression analyzed in 20-DAS plants under SD conditions. Relative mRNA levels are expressed as the ratios to β-tubulin transcript levels. cDNA was synthesized with random primers. Numbers above the white bars are the ratios of the expression levels in NILs for *FT2a-TO* (white bars) to those in *FT2a-HY* (Black bars)

Transposable elements (TEs) in introns often affect chromatin structure and modify RNA processing of the host gene and, therefore, influence its expression patterns [48–50]. Using qRT-PCR on cDNA synthesized with random primers, which targeted different regions, we analyzed *FT2a* expression in two sets of NILs for *FT2a-TO* and *FT2a-HY* grown in SD. In all three targeted regions (a–c in Fig. 6a), the *FT2a* transcript abundance was considerably lower (1/5 to <1/10) in NILs for *FT2a-TO* than in NILs for *FT2a-HY* (Fig. 6b).

**Fig. 7:**
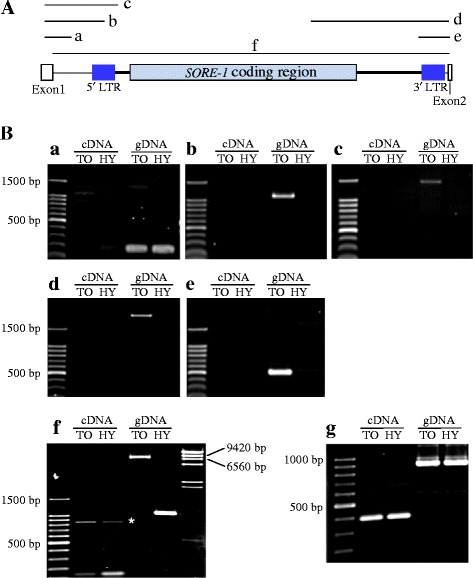
*FT2a* RNA processing in the first intron with *SORE-1* insertion. **a** a-f, Regions examined. **b** Semi-quantitative PCR analysis of *FT2a* expression in NILs (#115) for *FT2a-TO* (TO) and *FT2a-HY* (HY) in 20-DAS plants under SD conditions. cDNA was synthesized with random primers. *g*, amplification of the β-tubulin transcript. *, nonspecific amplification

To analyze *FT2a* RNA processing in *FT2a-TO*, we performed semi-quantitative RT-PCR on cDNAs synthesized with random primers. No amplicon was detected in regions a (from exon 1 to intron 1), b and c (from exon 1 to *SORE-1*), or d and e (from *SORE-1* to exon 2), although the expected amplicons were observed in PCR on genomic DNA of the NIL for *FT2a-TO* (Fig. 7). For region f (from exon 1 to exon 2), a fragment (~150 bp) was amplified in both NILs, although signal intensity was much higher in the NIL for *FT2a-HY* than in the NIL for *FT2a-TO*; as expected, genomic PCR produced fragments of 7,293 bp in the NIL for *FT2a-TO* and 1,064 bp in the NIL for *FT2a-HY* (Fig. 7b). These results suggest that intron 1 with the *SORE-1* insertion could be spliced out in the NIL for *FT2a-TO*.

**Fig. 8:**
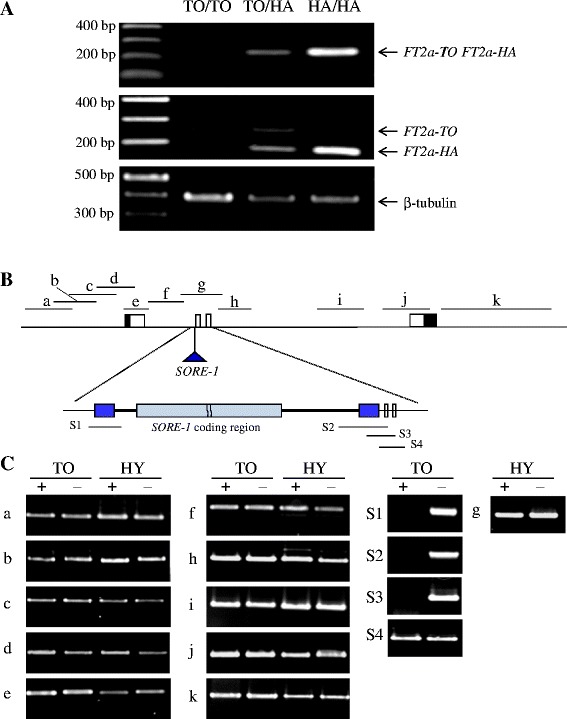
Transcript abundance of different alleles and DNA methylation in the *FT2a* genomic region. **a** Transcript abundance of different *FT2a* alleles assayed by allele-specific restriction digestion. **b** Diagram of the *FT2a* genomic region showing the position of *SORE-1* insertion. Amplicons were analyzed by semi-quantitative PCR after *Mcr*BC or mock digestion; the amplified regions are designated as *a* to *k* and S1 to S4. Exons, white; UTRs, black; LTRs of *SORE-1*, gray. **c** Genomic DNA from leaves of 20-day-old plants of NILs for *FT2a-TO* (TO) and *FT2a-HY* (HY) grown under SD conditions was digested with *Mcr*BC (+) or mock-digested (–) and amplified by PCR. Amplicons were visualized in agarose gels

Next, we examined *FT2a* expression in heterozygous siblings of NILs; this analysis was based on the fact that SNP #28 after the stop codon (Additional file 1) created a *Dde*I restriction site in *FT2a-HA*, but not in *FT2a-TO* and *FT2a-HY*. By performing RT-PCR and digesting the product with *Dde*I, expression of *FT2a-TO* can be distinguished from that of *FT2a-HA* in heterozygous plants. In the NILs-#5 for *FT2a-TO* and *FT2a-HA*, and its siblings, the *FT2a* transcript level was high in homozygotes for *FT2a-HA*, slightly lower in heterozygotes, and very low in homozygotes for *FT2a-TO* (Fig. 8a). Digestion of PCR products revealed that in heterozygotes, the transcript level of *FT2a-HA* was much higher than that of *FT2a-TO*. This difference suggests that the lower expression of *FT2a-TO* was caused by allele-specific transcriptional repression rather than sequence-specific RNA degradation of RNA silencing that decreases the levels of transcripts from both alleles.

We also evaluated the methylation of *FT2a-TO* and *FT2a-HY*. Methylation-dependent *Mcr*BC restriction digestions and mock digestions of genomic DNA were used to analyze cytosine methylation in NILs for *FT2a-TO* and *FT2a-HY*. Semi-quantitative PCR was performed using primers designed for each of the targeted regions to be singly amplified (Fig. 8b). There was no difference in PCR amplification of genomic regions a–f and h–k in the *Mcr*BC-digested and mock-digested samples in both NILs (Fig. 8c). In contrast, no amplicons were detected for regions S1–S3 (which include the LTRs of *SORE-1* and *FT2a* regions flanking the LTRs) after *Mcr*BC digestion in the NIL for *FT2a-TO*, although fragments of expected sizes were amplified from mock-digested DNA. PCR on both *Mcr*BC-digested and mock-digested DNAs produced the expected amplicons in region S4 (which did not include the LTR sequence) of the NIL for *FT2a-TO* and in genomic region g (which did not contain *SORE-1*) of the NIL for *FT2a-HY*. Taken together, these data indicate that *SORE-1* was highly methylated, but methylation appeared not to extend to the *FT2a* genomic region flanking *SORE-1*. The same result was obtained for plants grown in LD (data not shown), which indicates that lower mRNA level of *FT2a-TO* is associated with *SORE-1* methylation in both SD and LD conditions.

## Discussion

### Maturity gene *E9* is *FT2a*

Flowering time in the F_2_ and F_3_ progeny of a cross between TO and HA co-segregated with the alleles at the *E1* and *E9* loci. Fine mapping delimited *E9* to a 40.1-kb region that contained three genes, including *FT2a*, a soybean ortholog of *FT* (Fig. 2). Sequencing and expression analysis suggested that *FT2a* is the most likely candidate for *E9*, and delayed flowering due to *e9* is most likely caused by the reduced *FT2a* transcript abundance. Despite sequence identity in the coding regions, we detected several SNPs and indels of 4–43 bp in the promoter and 5′ UTR among cultivars and accessions tested; this is consistent with a previous report [42]. However, expression analysis of NILs and photoperiod-insensitive accessions carrying different *FT2a* alleles revealed that the polymorphisms in the promoter and 5′ UTR were not responsible for different *FT2a* expression levels (Figs. 4 and 5). TO also differed from HA and HY by a SNP and a *SORE-1* insertion in the first intron, of which the latter was solely associated with the *FT2a* expression levels (Fig. 5). Thus, our study reveals that the insertion of *SORE-1* attenuated *FT2a* expression and delayed flowering. The soybean genome possesses a total of ten *FT* orthologs, among which six retain the *FT* function and can promote flowering of *Arabidopsis ft* mutants [22, 23] or Col-0 [24, 25]. All of the six homologs could therefore function as potential floral inducers in soybean, although only two of them, *FT2a* and *FT5a*, have been extensively characterized in studies of molecular mechanisms of flowering [15, 19, 22, 24, 26–28, 40, 43]. This study demonstrates that different levels of *FT2a* expression directly regulate natural variation in flowering time in soybean.

### Factors responsible for attenuation of *FT2a* expression

Plant TEs inserted in introns may affect RNA processing [48, 49] and render their host genes susceptible to short interfering RNA (siRNA)-mediated silencing [50]. Our results show that the first intron (including *SORE-1*) is spliced out, because no primary RNA transcripts that would cover *FT2a* exons and *SORE-1* were detected while the spliced products were detected (Fig. 7). Thus, *SORE-1* insertion did not markedly interfere with *FT2a* RNA processing.

We found that the reduction in *FT2a-TO* transcript abundance was caused by allele-specific transcriptional repression due to the insertion of *SORE-1*, the LTRs and adjacent sequences of which were highly methylated (Fig. 8). Therefore, epigenetic mechanisms likely account for the reduction in *FT2a-TO* transcript levels. RNA-directed DNA methylation or the resulting chromatin modifications regulate gene expression by interfering with transcription factor binding, leading to different expression profiles for different transcription factors [50–52]. PLACE analysis detected two *cis*-elements, RBCSCONSENSUS and ARR1AT, in the region flanking the *SORE-1* integration site in the first intron. However, the functions of the two elements in *FT2a* expression are unclear. A further test is thus needed to determine the functions of the two *cis*-elements or nearby unknown elements in the regulation of *FT2a* expression and whether *SORE-1* insertion interrupts binding of a transcriptional factor(s) to these *cis*-elements.

Methylation-mediated gene repression by intronic TEs is well characterized in *Arabidopsis FLOWERING LOCUS C* (*FLC*), which encodes a transcription factor containing a MADS domain that inhibits *FT* expression [53, 54]. In Col-0, the functional *FLC* allele is highly expressed in the presence of *FRIGIDA* and causes extremely late flowering [54]. In contrast, in ecotype Landsberg *erecta* (L*er*), the *FLC* allele has a 1,224-bp non-autonomous Mutator-like TE in intron 1 and is expressed at low levels due to its transcriptional silencing through histone H3-K9 methylation, which is triggered by siRNA generated from homologous TEs [50]. *FLC*-L*er*, however, can still be regulated by genes in the autonomous flowering pathway and by genes involved in vernalization, because the TE insertion does not affect the transcription factor–binding sites in intron 1 [50]. Similarly to the *FLC*-L*er* allele, the expression of *FT2a-TO* is repressed due to epigenetic modification caused by the insertion of *SORE-1* in intron 1. However, *FT2a-TO* expression was still higher in SD than in LD (Fig. 3). Virus-induced silencing of *E1-like* genes (repressors of *FT2a* and *FT5a*) lowers photoperiod sensitivity of TO by up-regulating the expression of both *FT2a* and *FT5a* [28]. The regulation of *FT2a* expression by *E1-like* and other genes involved in photoperiod responses is thus retained in *FT2a-TO* plants. The *FT2a-TO* allele may thus be involved in flowering as a leaky allele, not a dysfunctional allele.

### Origin and adaptive role of the *e9* allele

*SORE-1* was first detected in a recessive allele at the *E4* locus encoding phytochrome A; its insertion in the first exon caused a premature stop codon and resulted in a dysfunctional truncated protein [21]. DNA marker analysis revealed that the *e4* allele with the *SORE-1* insertion is present mainly in landraces from northern Japan [46], although it has been used in breeding of photoperiod-insensitive cultivars in high-latitude regions of other countries [29]. This insertion in the *E4* gene may thus have played an adaptive role in expanding the areas of soybean cultivation to higher latitudes. Our preliminary survey of the insertion of *SORE-1* in the *FT2a* allele suggests that *FT2a-TO* is a region-specific allele detected in only a few local varieties established in Sakhalin and northern Hokkaido among photoperiod-insensitive landraces and cultivars having the *e4* allele with the *SORE-1* insertion. Therefore, the insertion of *SORE-1* in the first intron of *FT2a-TO* may be of recent origin.

Our preliminary survey also suggests that the cultivar Toshidai-7910, introduced from Sakhalin with Karafuto 1, was the source of the *FT2a* allele with the *SORE-1* insertion in TO [44]. Similar to TO, both Toshidai-7910 and Karafuto 1 have a null allele at the *E1* locus, but, unlike TO, they have recessive alleles at *E3* and *E4* ([18] and this study). This is a maturity genotype that permits extremely early flowering and maturation and enables seed production in cold climates with a limited frost-free season. Because *FT2a* and *FT5a* control flowering redundantly [24, 27], the *e9* (*FT2a-TO*) allele could have been selected in the presence of functional *FT5a* because it maintains vegetative growth. It is thus another example of the adaptive role of *SORE-1* insertion as indicated by Kanazawa et al. [46]. The *e9* allele may also be useful for developing cultivars adapted to a shorter photoperiod in low-latitude environments where flowering is strongly promoted. In such environments, a longer vegetative phase, a so-called long-juvenile trait, is desirable. A leaky allele similar to *e9* may be useful for reducing the transcript levels of *FT2a* under SD conditions, in addition to long-juvenile genes reported so far, such as *E6* [9] and *j* [13]. A further study is needed to evaluate the adaptive significance of *e9* under SD conditions.

## Conclusions

The present study revealed that the soybean maturity gene *E9* is *FT2a*, an ortholog of *Arabidopsis FT*, and that its recessive allele delays flowering through lower transcript abundance. *FT2a* is thus directly involved in the natural variation in flowering time in soybean. The attenuation of *FT2a* expression is caused by allele-specific transcriptional repression caused by the insertion of *SORE-1* in the first intron. The recessive *e9* allele is a leaky allele; its regulation by other genes involved in photoperiod response is retained. It may thus maintain vegetative growth in early-flowering genetic backgrounds, and also be useful as a long-juvenile allele in cultivar development in low-latitude regions, where flowering is strongly promoted.

## Methods

### Plant materials

Three early-maturing soybean cultivars were used in this study: the Canadian cultivar Harosoy (L58-266; HA) and two Japanese cultivars, Toyomusume (TO) and Hayahikari (HY). HA and TO have the *e2*/*e2*, *E3*/*E3*, and *E4*/*E4* allele composition; HA has a hypomorphic *e1-as* allele, whereas TO lacks the genomic region (~130 kb) containing the entire *E1* gene (*e1-nl* allele) [18]. HY has the *E1*/*E1*, *e2*/*e2*, *e3*/*e3*, and *e4*/*e4* allelic composition and is photoperiod-insensitive [18]. We developed four sets of F_6_ near-isogenic lines (NILs) for the *E9* gene from the progeny of F_5_ heterozygous plants: two sets from the cross between TO and HA and two from the cross between TO and HY. All NILs had the same genotype (*e1-nl*/*e1-nl*, *e2*/*e2*, *E3*/*E3*, and *E4*/*E4*) as TO. The breeding line TK780 and the wild soybean accession Hidaka 4 were used for sequencing because they were parents of the recombinant inbred population used for identification of the *E9* gene [12, 14]. Five photoperiod-insensitive accessions (Karafuto 1, Gokuwase-Kamishunbetsu, Nawiko, Heihe 13, and Kitamusume) were used for expression analyses.

### Segregation analysis and fine mapping of the *E9* gene

Seeds of the F_2_ population (*n* = 82) and both parents, TO and HA (*n* = 10), were sown in paper pots on 25 May 2012, and 10 days later seedlings were transplanted into soil at an experimental farm of Hokkaido University, Sapporo (43°07′N, 141°35′E). The 82 F_2_ plants were genotyped with a DNA marker at the *E1* locus [18] and its flanking SSR marker [15], and 16 plants homozygous for *e1-nl* and 16 plants homozygous for *e1-as* were selected for the progeny test. Seeds of each F_2_ plant were sown on 25 May 2013, and 10 days later 20 seedlings were transplanted into the same field. The number of days from sowing to the first flower opening (R1) [55] of each plant was recorded. For fine-mapping of the *E9* gene, a total of 300 seeds from two heterozygous F_3_ plants derived from the same F_2_ family (#41) were genotyped for SSR and indel markers flanking this gene. Eight recombinants between markers were cultivated in a glasshouse during winter, and the seeds produced were used for the progeny test during summer (sowing date: 15 May 2014, *n* = 20).

### DNA marker analysis

Total DNA was extracted from trifoliate leaves as described [56] and from seeds as described [15]. Sixty-one SSR markers mapped on the consensus map (SOYBASE; http://soybase.org/sbt/) [57, 58] and located in the genomic regions where orthologs to *Arabidopsis* flowering genes are clustered [4] were chosen for tests of their association with flowering time. Data analysis in association tests were performed by one-way analysis of variance. The following DNA markers flanking *E9* were used for fine mapping: nine SSR markers available in the genomic sequence database of Williams 82 (Gmax v. 2.0; http://www.phytozome.net/soybean) [44] and three indel markers [12]. Each PCR contained 30 ng of total genomic DNA as template, 1 μl of each primer (10 μM) and dNTP (2.5 mM), 0.5 μl of *ExTaq* polymerase, and 2.5 μl of 10× *ExTaq* buffer (Takara, Otsu, Japan) in a total volume of 25 μl; amplification conditions were 35 cycles at 94 °C for 30 s, 56 °C to 60 °C (depending on the primers used) for 30 s, and 72 °C for 30–90 s. PCR products were separated by electrophoresis in 10.5 % (w/v) polyacrylamide gels (for SSR markers) or 1 % agarose gels (for indels), stained with ethidium bromide, and visualized under UV light.

### Expression analysis

Plants were grown in growth cabinets under SD (12 h) or LD (18 h) conditions at 24 °C. Fully developed trifoliate leaves of four 20-day-old plants were sampled as a bulk at Zeitgeber time 3 to compare the expression levels of *FT2a* between plants with different alleles or every three hours to determine the diurnal patterns in TO and HA. Leaves were immediately frozen in liquid N_2_, and stored at −80 °C. Total RNA was isolated from frozen tissues by lithium chloride precipitation according to Napoli et al. [59], except that DNase I (Takara Bio, Otsu, Japan) was used to remove genomic DNA. cDNA was synthesized from 1 μg of total RNA using an oligo (dT) 18 primer or random primer cocktail (Takara Bio, Otsu, Japan) according to Dwiyanti et al. [60]. *FT2a* transcript levels were determined by semi-quantitative RT-PCR or quantitative real-time PCR (qRT-PCR). Each of semi-quantitative RT-PCR contained 0.5 μg of cDNA (0.1 μg for total genomic DNA used as a control) as template, 1 μl of each primer (10 μM) and dNTP (2.5 mM), 0.5 μl of *ExTaq* polymerase, and 2.5 μl of 10 × *ExTaq* buffer in a total volume of 25 μl; amplification conditions were 33 cycles at 94 °C for 30 s, 60 °C or 64 °C for 30 s (depending on the primers used), and 72 °C for 30 s–8 m (depending on the sizes of amplified fragments). The qRT-PCR mixture (20 μL) contained 0.05 μL of the cDNA synthesis reaction, 5 μL of 1.2 μM primer premix, and 10 μL SYBR Premix *ExTaq* Perfect Real Time (Takara, Otsu, Japan). A CFX96 Real-Time System (Bio-Rad Laboratories Japan, Tokyo, Japan) was used. The PCR cycling conditions were 95 °C for 3 min followed by 35 cycles of 95 °C for 10 s, 60 °C for 30 s, 72 °C for 25 s and 78 °C for 2 s. Fluorescence was quantified before and after the incubation at 78 °C to monitor the formation of primer dimers. The mRNA for β-tubulin was used as a control. A reaction mixture without reverse transcriptase was also used as a control to confirm the absence of genomic DNA contamination. Amplification of a single DNA fragment was confirmed by melting curve analysis and gel electrophoresis of the PCR products. Averages and standard errors of relative expression levels were calculated from PCR results for three independently synthesized cDNAs. Primer sequences used in expression analyses are listed in Additional file 3.

### Sequencing analysis of *FT2a* and *SORE-1*

cDNAs from the three cultivars, TK780 and Hidaka 4 were used to sequence the *FT2a* coding regions. Each of the two *FT2a* genomic regions (the 5′ upstream region and the genic to 3′ downstream region) was divided into three parts, which were amplified from total DNA with KOD FX polymerase (Toyobo Life Science, Osaka, Japan) and sequenced. Genome walking with a BD Genome-Walker Universal kit (Takara Clontech, Otsu, Japan) was used to sequence the first intron of TO, in which *SORE-1* was inserted. According to the manufacturer’s instructions, we constructed four kinds of genomic libraries by digesting total DNA from TO in separate reactions with four blunt-end endonucleases (*Dra*I, *Eco*RV, *Puv*II, and *Stu*I) and ligating the ends of the digested DNA to an adaptor sequence. Nested PCR was performed for each library using adaptor primers and gene-specific primers. The inserted *SORE-1* was then amplified with the forward primer in intron 1 and the reverse primer in intron 2, and the resultant amplicon was used for PCR amplification of each of five divided regions of *SORE-1* to obtain the whole sequence (Additional file 4). The amplified fragments were sequenced directly or were first cloned into a pGEM-T Easy vector (Promega, Madison, WI, USA) and then sequenced. Sequence analysis was performed by using a BigDye Terminator v. 3.1 Cycle Sequencing kit and an ABI PRISM 3100 Avant Genetic Analyzer (Applied Biosystems Japan, Tokyo, Japan) according to the manufacturer’s instructions. A BLAST search of the NCBI genome database and PLACE [47] analysis were carried out to detect sequences homologous to the fragment identified by genome-walking and possible *cis*-elements in the first intron of *FT2a*. Primer sequences used in genome sequencing are listed in Additional file 4.

### Genotyping for DNA polymorphisms in the *FT2a* genomic region and maturity loci

DNA markers were developed to detect a 10-bp deletion in the 5′ UTR and the insertion of SNP #17 and *SORE-1* in the first intron. For the 10-bp deletion, the primers 5′-GGAATCGAGGCTATTGACTA-3′ and 5′-CTTCCACTAGGCATGGGATA-3′ were used. For *SORE-1*, two forward primers, 5′-GCTCTCTCTCTTCCACTCTCTAGATGG-3′ (in the long terminal repeat [LTR] of *SORE-1*) and 5′-ACCCTCTCAAGTGGACATGT-3′ (in the first *FT2a* intron), and the common reverse primer 5′-CTAGGTGCATCGGGATCAAC-3′ (in the second *FT2a* exon) were used. To identify the SNP, a dCAPS marker was developed: PCR was performed with the primers 5′-TTCAAACAATCTCATAATTATGAGT-3′ and 5′-TAATAGTAGTATGGATGGTCAAA-3′, and the amplified products were digested with *Hinf*I. The PCR reaction and detection of amplified fragments were performed as described above. The genotyping for the *E1*, *E3*, and *E4* loci was performed using allele-specific DNA markers as described [18, 29]. Primers, PCR conditions and expected fragment sizes are presented in Additional file 5.

### Methylation analysis

Genomic DNA was extracted from trifoliate leaves of 20-day-old plants of NILs for the *FT2a-TO* and *FT2a-HY* alleles, grown under SD. DNA samples were digested with *Mcr*BC (Takara Bio, Otsu, Japan). Digested and undigested samples were used for semi-quantitative PCR amplification of different regions of *FT2a* genomic and *SORE-1* regions. Primer sequences used in methylation analyses are listed in Additional file 6.

### Availability of supporting data

All supporting data can be found within the manuscript and its additional files. *FT2a* genomic sequences of Harosoy, Toyomusume and Hayahikari were deposited in the DNA Data Bank of Japan (DDBJ) under the accession numbers LC086649, LC086650 and LC086651, respectively.

## Additional files

Additional file 1:
**DNA polymorphisms detected in the** ***FT2a*** **genomic region in four soybean cultivars or breeding lines and a wild soybean accession.** (PDF 31 kb) Additional file 2:
**Diurnal expression patterns of** ***FT2a.*** (PDF 77 kb) Additional file 3:
**Sequences of primers used in expression and RNA processing analyses of** ***FT2a.*** (PDF 88 kb) Additional file 4:
**Genomic positions and sequences of primers used in sequencing of the** ***FT2a*** **genomic region and** ***Ty1/copia-lik*** **e retrotransposon,** ***SORE-1.*** (PDF 130 kb) Additional file 5:
**Primers, PCR conditions and amplified fragment sizes for allele-specific DNA markers at maturity loci.** (PDF 108 kb) Additional file 6:
**Sequences of primers used in methylation analysis of** ***FT2a.*** (PDF 129 kb)

## Abbreviations

QTL: Quantitative trait locus
NIL: Near-isogenic line
RIL: Recombinant inbred line
PCR: Polymerase chain reaction
qRT-PCR: Quantitative real-time PCR
dCAPS: Derived cleaved amplified polymorphic sequence
*FT*: *FLOWERING LOCUS T*
DAS: Days after sowing
DAE: Days after emergence
TE: Transposable element
