# Supplementary material for: A recessive allele for delayed flowering at the soybean maturity locus E9 is a leaky allele of FT2a, a FLOWERING LOCUS T ortholog
Source: BMC Plant Biol. 2016 Jan 19;16:20. doi: 10.1186/s12870-016-0704-9 (PMC4719747; doi:10.1186/s12870-016-0704-9)
Supplement: Additional file 1: — DNA polymorphisms detected in the FT2a genomic region in four soybean cultivars or breeding lines and a wild soybean accession. (PDF 31 kb) [file 12870_2016_704_MOESM1_ESM.pdf]

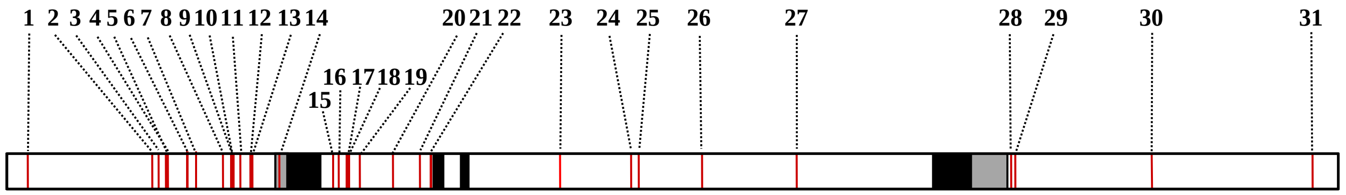

	1	2	3	4	5	6	7	8	9	10	11	12	13	14
	Promoter													5' UTR
	-1544	-772	-731	-698	-694	-571	-544	-402	-379	-375	-349	-279	-274	-47
Toyomusume	A	T	43 bp	A	T	G	AT	G	G	G	T	-	-	-
TK780	A	T	43 bp	A	T	G	AT	G	G	G	T	-	-	-
Hayahikari	T	T	43 bp	A	T	G	AT	G	G	G	T	-	GATC	10 bp
Hidaka 4	A	A	-	T	-	T	AT	A	G	G	C	-	-	10 bp
Harosoy	A	A	-	T	T	G	-	G	A	A	T	G	-	10 bp

	15	16	17	18	19	20	21	22
	Intron I							
	294	315	398	403	475	710	965	988
Toyomusume	G	C	C	T	T	T	SORE-1	T
Hayahikari	G	C	A	T	C	C	-	C
Harosoy	T	T	A	A	T	T	-	T

	23	24	25	26	27	28	29	30	31
	Intron II					3' downstream region			
	2150	2604	2648	3022	3783	4606	4628	5300	6630
Toyomusume	(C)$_{10}$ A (C)$_{8}$ (A)$_{6}$	(T)$_{11}$ T	(AT)$_{8\text{-}11}$	(A)$_{10}$	-	A	(A)$_{6}$	G	A
Hayahikari	(C)$_{10}$ A (C)$_{8}$ (A)$_{6}$	(T)$_{10}$ A	(AT)$_{18}$	(A)$_{10}$	9 bp	A	(A)$_{7}$	G	G
Harosoy	(C)$_{16}$ (A)$_{9}$	(T)$_{11}$ A	(AT)$_{27\text{-}30}$	(A)$_{9}$	-	G	(A)$_{6}$	T	A

43 bp-indel CCCCTGCTAATCTTCTTCCTCCTCACACATTGCTTCTCACCCC
10 bp-indel GAAAGCATAA
SORE-1 *Ty1/copia-like* retrotransposon (6,224 bp)
9 bp-indel AAAGAAAAA

Additional file 1. DNA polymorphisms detected in the *FT2a* genomic region for four soybean cultivars or breeding line and a wild soybean accession.
