# Supplementary material for: A recessive allele for delayed flowering at the soybean maturity locus E9 is a leaky allele of FT2a, a FLOWERING LOCUS T ortholog
Source: BMC Plant Biol. 2016 Jan 19;16:20. doi: 10.1186/s12870-016-0704-9 (PMC4719747; doi:10.1186/s12870-016-0704-9)
Supplement: Additional file 2: — Diurnal expression patterns of FT2a. (PDF 77 kb) [file 12870_2016_704_MOESM2_ESM.pdf]

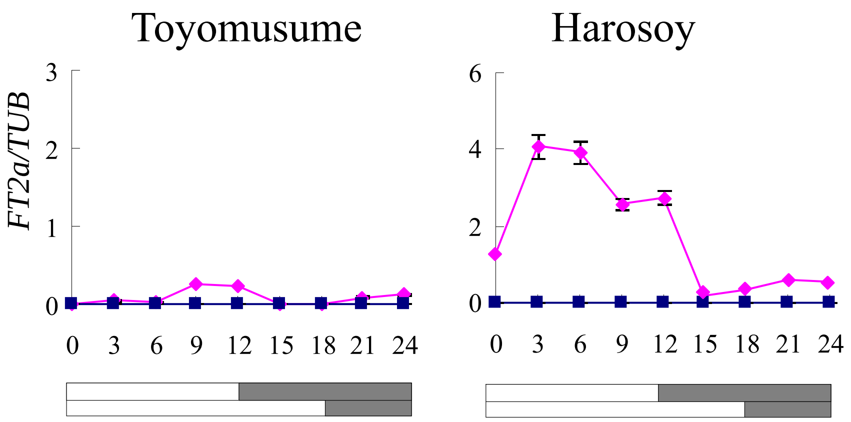

Additional file 2. Diurnal expression patterns of *FT2a*.

Transcript abundances of *FT2a* were analyzed in SD (pink line) and LD (blue line) conditions.
