# Supplementary material for: A recessive allele for delayed flowering at the soybean maturity locus E9 is a leaky allele of FT2a, a FLOWERING LOCUS T ortholog
Source: BMC Plant Biol. 2016 Jan 19;16:20. doi: 10.1186/s12870-016-0704-9 (PMC4719747; doi:10.1186/s12870-016-0704-9)
Supplement: Additional file 3: — Sequences of primers used in expression and RNA processing analyses of FT2a. (PDF 88 kb) [file 12870_2016_704_MOESM3_ESM.pdf]

Area targeted		Primer sequence (5' – 3')	Annealing tempreture	Extension time
FT2a-a	F	TAAACTAGTGTGCACACTATCCC	60º C	25 s
	R	TATAGAAGTTCCTGAGGTCATCACCA		
FT2a-b	F	GGGGAGTAATTGGGGATGTATTGG	60º C	25 s
	R	AAACTAGCCCCTGTTGTTGC		
FT2a-c	F	GGATTGCCAGTTGCTGCTGT	60º C	25 s
	R	GAGTGTGGGAGATTGCCAAT		
tubulin	F	GAGAAGAGTATCCGGATAGG	60º C	25 s
	R	GAGCTTGAGTGTTCGGAAAC		
a	F	GGGGATAATTGGGGATGTATTGG	60º C	20 s
	R	CAAAAAGAGTACTTGGACAA		
b	F	GGGGAGTAATTGGGGATGTATTGG	60º C	1 min 20 s
	R	CACCATCTAGAGAGTGGAAGAGAGAG		
c	F	GGGGAGTAATTGGGGATGTATTGG	60º C	1 min 36 s
	R	ACCAAGAATAAACATTGGGT		
d	F	AACTTCATGATCAAGGTGCT	60º C	2 min
	R	AAACTAGCCCCTGTTGTTGC		
e	F	GCTCTCTCTCTTCCACTCTCTAGATGG	60º C	40 s
	R	AAACTAGCCCCTGTTGTTGC		
f	F	GGGTAAATATCGGTGGTGATGAC	64º C	8 min
	R	AAACTAGCCCCTGTTGTTGC		

Additional file 3. Sequences of primers used in expression and RNA processing analyses of *FT2a*. Targeted *FT2a* areas are shown in Figure 6 (a to c) and Figure 7 (a to f).
