# Supplementary material for: A recessive allele for delayed flowering at the soybean maturity locus E9 is a leaky allele of FT2a, a FLOWERING LOCUS T ortholog
Source: BMC Plant Biol. 2016 Jan 19;16:20. doi: 10.1186/s12870-016-0704-9 (PMC4719747; doi:10.1186/s12870-016-0704-9)
Supplement: Additional file 4: — Genomic positions and sequences of primers used in sequencing of the FT2a genomic region and Ty1/copia-lik e retrotransposon, SORE-1. (PDF 130 kb) [file 12870_2016_704_MOESM4_ESM.pdf]

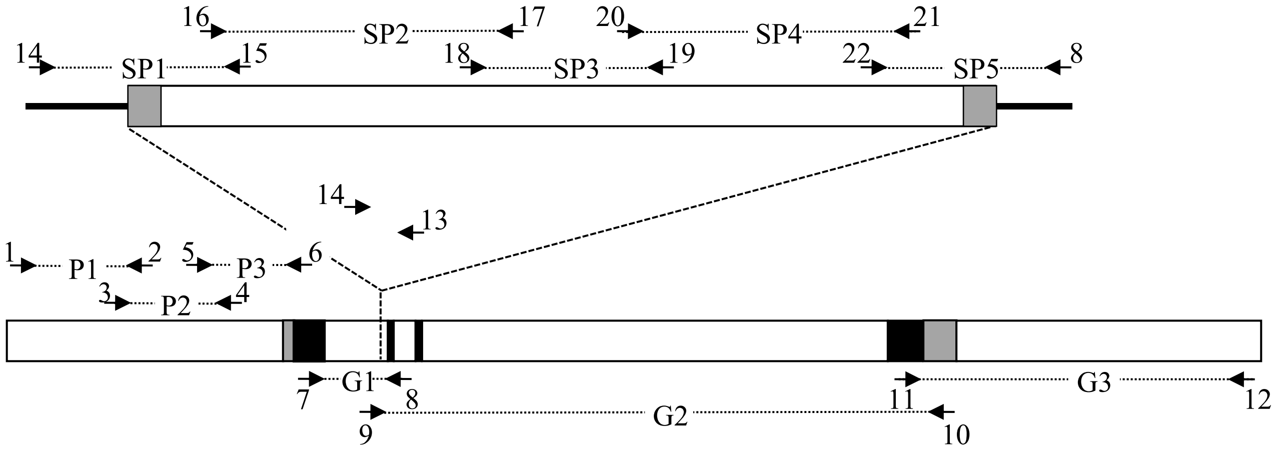

Area targeted	Primer sequence (5' – 3')		Annealing tempreture	Extension time
P1	1	AACAAAGAGAGAGAGAGAGGAAC	57 °C	1 min
	2	GCTTTTCCCTCTCAATTAAGAATC		
P2	3	CCTACAAACAAAATTGATGATGTTTCTAG	57 °C	1 min
	4	TTCAAACATTTTCACATCCCTTCC		
P3	5	AGTCATACATTACGGAATCTAGTT	57 °C	45 s
	6	CTTCCACTAGGCATGGGATA		
G1	7	GGGGAGTAATTGGGGATGTATTGG	56 °C	1 min 20 s
	8	CTAGGTGCATCGGGATCAAC		
G2	9	TATATTCTTAGTGCAATCGAGGATCATTAG	62 °C	4 min
	10	GGGAGATTGCCAATTAATATTTCTGAAA		
G3	11	ATTTCGTCAACTGGGTAGGGAG	55 °C	3 min
	12	GCAGATTCTCATAACGTCGGAG		
Genomic walker primer	13	AGTCACCAACCTGACACATTGAGAGAAGAT	63 °C	4 min
Full SORE-1	14	ACCCTCTCAAGTGGACATGT	62 °C	8 min 30 s
	8	CTAGGTGCATCGGGATCAAC		
SP1	14	ACCCTCTCAAGTGGACATGT	60 °C	1 min 30 s
	15	CGATAACTCAGCTGCTGTCT		
SP2	16	CCTTAAATTTAAACGGTGGC	55 °C	2 min
	17	ACATGATTGGCATTCCACAA		
SP3	18	CAGGATCATTTTCAGCTCCA	55 °C	1 min 30 s
	19	TTCCTCTATGTTATGGCACC		
SP4	20	CATCCAATGCTTCGTATTTG	55 °C	1 min 30 s
	21	GACATAGATTATGCTATAAGG		
SP5	22	AACTTCATGATCAAGGTGCT	60 °C	2 min
	8	CTAGGTGCATCGGGATCAAC		

Additional file 4. Genomic positions and sequences (5′ – 3′) of primers used in sequencing of the *FT2a* genomic region and *Ty1/copia-like* retrotransposon, *SORE-1*. Primer 13 was used for nested PCRs for genome libraries of Toyomusume. The PCR products amplified with primers 14 and 8 were used as temperate in PCR amplifications for the *SORE-1* and its flanking region (SP1 to SP5).
