# Supplementary material for: A recessive allele for delayed flowering at the soybean maturity locus E9 is a leaky allele of FT2a, a FLOWERING LOCUS T ortholog
Source: BMC Plant Biol. 2016 Jan 19;16:20. doi: 10.1186/s12870-016-0704-9 (PMC4719747; doi:10.1186/s12870-016-0704-9)
Supplement: Additional file 5: — Primers, PCR conditions and amplified fragment sizes for allele-specific DNA markers at maturity loci. (PDF 108 kb) [file 12870_2016_704_MOESM5_ESM.pdf]

Locus / Alleles	Primer sequence (5' – 3')		Annealing tempreture	Size of fragment (bp)	
E1 [1]	F	CACTCAAATTAAGCCCTTTCA	60 ºC	*e1-as*	222
e1-as / *e1-nl*	R	TTCATCTCCTCTTCATTTTTGTTG		*e1-nl*	no products
E3 [2]	E3_08557FW	TGGAGGGTATTGGATGATGC	58 ºC	*E3*	558
E3 / *e3-tr*	E3Ha_1000RV	CGGTCAAGAGCCAACATGAG		*e3-tr*	275
	e3tr_0716RV	GTCCTATACAATTCTTTACGACG			
E4 [3]	F	AGACGTAGTGCTAGGGCTAT	55 ºC	*E4*	1,229
E4 / *e4-SORE-1*	R2	GCATCTCGCATCACCAGATCA		*e4-SORE-1*	837
	R3	GCTCATCCCTTCGAATTCAG			

Additional file 5. Primers, PCR conditions and expected amplified fragment sizes for allele-specific DNA markers.

1, 3) Xu et al. [29], 2) Tsubokura et al. [18]
