# Supplementary material for: A recessive allele for delayed flowering at the soybean maturity locus E9 is a leaky allele of FT2a, a FLOWERING LOCUS T ortholog
Source: BMC Plant Biol. 2016 Jan 19;16:20. doi: 10.1186/s12870-016-0704-9 (PMC4719747; doi:10.1186/s12870-016-0704-9)
Supplement: Additional file 6: — Sequences of primers used in methylation analysis of FT2a. (PDF 129 kb) [file 12870_2016_704_MOESM6_ESM.pdf]

Area targeted		Primer sequence (5' − 3')	Annealing tempreture	Extension time
a	F	TGAAGTCTCTGAACATGCACGC	58 ºC	1 min
	R	CACCTTTATATATTCCTCATATCTGTC		
b	F	AAAGGTGAAATATATATTGTTGG	64 ºC	40 s
	R	CTTCCACTAGGCATGGGATA		
c	F	TATAGAAATTTCAGAAATTTCTAGCAGCG	60 ºC	40 s
	R	TTCAAACATTTTCACATCCCTTCC		
d	F	GATTCTTAATTGAGAGGGAAAAGC	60 ºC	40 s
	R	AAAGGAGCAGCAAAACGCTA		
e	F	GGAATCGAGGCTATTGACTA	58 ºC	1 min
	R	CAAAAAGAGTACTTGGACAA		
f	F	ACCAAGCTAGAATAATTTTTGTGAG	60 ºC	40 s
	R	TGAGTGGTGGGGTTTTCTTT		
g	F	ACCCTCTCAAGTGGACATGT	64 ºC	40 s
	R	AAACTAGCCCCTGTTGTTGC		
h	F	ACACGCCATGAATGCAAACA	64 ºC	40s
	R	GCAAGCAAACATTGTGCGTT		
i	F	CCGAATTCTTAAAGTGCATGCA	64 ºC	40s
	R	GCCAGAAACACTCATACATACT		
j	F	GGACAGCAGTAGTAGGACCA	64 ºC	1 min 35 s
	R	GGGAGATTGCCAATTAATATTTCTGAAA		
k	F	TGATAGTCTATGGGATTGTGC	64 ºC	40 s
	R	CCAAGCTTCCAACCGTGAAA		
S1	F	AATATAGTAGTAGTTTCTAGCTT	60 ºC	40 s
	R	ACCAAGAATAAACATTGGGT		
S2	F	TATATCATGATGCCTGTGGG	64 ºC	1 min 35 s
	R	CTAGGTGCATCGGGATCAAC		
S3	F	GCTCTCTCTCTTCCACTCTCTAGATGG	64℃	40 s
	R	AAACTAGCCCCTGTTGTTGC		
S4	F	CCAACTTGATTTCATTTCAT	64 ºC	1 min 35 s
	R	GCAAGCAAACATTGTGCGTT		

Additional file 6. Sequences of primers used in methylation analysis of *FT2a*. Targeted areas are presented in Figure 8.
